# Susceptibility of Clinical Isolates of *Burkholderia pseudomallei* to a Lipid A Biosynthesis Inhibitor

**DOI:** 10.4269/ajtmh.16-0858

**Published:** 2017-04-24

**Authors:** Sineenart Sengyee, Natnaree Saiprom, Suporn Paksanont, Direk Limmathurotsakul, Vanaporn Wuthiekanun, Narisara Chantratita

**Affiliations:** 1 Department of Microbiology and Immunology, Faculty of Tropical Medicine, Mahidol University, Bangkok, Thailand;; 2 Mahidol-Oxford Tropical Medicine Research Unit, Faculty of Tropical Medicine, Mahidol University, Bangkok, Thailand;; 3 Department of Tropical Hygiene, Faculty of Tropical Medicine, Mahidol University, Bangkok, Thailand

## Abstract

*Burkholderia pseudomallei* is the causative agent of melioidosis, a serious infection associated with high mortality and relapse. Current antimicrobial therapy using ceftazidime (CAZ) is often ineffective. Inhibitors of LpxC, the enzyme responsible for lipid A biosynthesis, have potential antimicrobial activity against several Gram-negative bacteria in vivo, but their activity against *B. pseudomallei* is unclear. Herein, we investigated the susceptibility of *B. pseudomallei* clinical isolates to LpxC-4, an LpxC inhibitor, and LpxC-4 in combination with CAZ. Time-kill assays for bactericidal activity were conducted for *B. pseudomallei* K96243, revealing growth inhibition and bactericidal effect at LpxC-4 concentrations of 2 μg/mL and 4 μg/mL, respectively. No significant synergistic effect was observed with the combination of LpxC-4 and CAZ. LpxC-4 susceptibility was tested on three groups of clinical isolates:1) CAZ- and trimethoprim–sulfamethoxazole (SXT)–susceptible (*N* = 71), 2) CAZ-resistant (*N* = 14), and 3) SXT-resistant (*N* = 23) isolates, by broth microdilution. The minimum concentration of LpxC-4 required to inhibit the growth of 90% of organisms was 2 μg/mL for all isolates. The median minimum inhibitory concentration of both the CAZ/SXT-susceptible and CAZ-resistant groups was 1 μg/mL (interquartile range [IQR] = 1–2 μg/mL), compared with 2 μg/mL (IQR = 2–4 μg/mL) for the SXT-resistant group. Cell morphology was observed after drug exposure by immunofluorescent staining, and a change from rod-shaped to cell wall–defective spherical cells was observed in surviving bacteria. LpxC-4 is a potent bactericidal agent against *B. pseudomallei* and warrants further testing as a new antibiotic to treat melioidosis.

## INTRODUCTION

*Burkholderia pseudomallei* is an environmental Gram-negative bacillus that causes the serious infection, melioidosis. The disease is highly endemic and a major cause of community-acquired infection in tropical and subtropical regions. Patients are primarily infected with *B. pseudomallei* by percutaneous inoculation, inhalation, and ingestion.^1^ A recent study estimated that *B. pseudomallei* causes 165,000 cases of melioidoisis per year worldwide, of which 89,000 patients are predicted to die.^2^ Melioidosis is associated with a high mortality rate, which can be up to 40% even with appropriate treatment.^1^ There is currently no vaccine available. Most melioidosis patients have underlying diseases and risk factors that include diabetes, pulmonary disease, renal disease, thalassemia, alcohol use, glucocorticoid therapy, and cancer. The clinical manifestations range from an acute septic form to chronic infection. Bacteremia, pneumonia, genitourinary infection, skin infection, and abscesses in several organs are common features of the disease.^1^

Melioidosis is difficult to treat because *B. pseudomallei* is resistant to several classes of antimicrobial agents including cephalosporins, macrolides, penicillins, polymyxins, and aminoglycosides.^1^ Delayed therapy of patients can be fatal because empirical antibiotic treatment used for bacterial sepsis does not treat *B. pseudomallei* infection. In Thailand, the recommended antimicrobial treatment of melioidosis consists of 10–14 days of ceftazidime (CAZ) administered intravenously followed by oral eradication therapy, with trimethoprim–sulfamethoxazole (SXT) for 3–6 months.^1^^,^^3^ Despite the rate of antimicrobial resistance testing to CAZ and SXT in vitro being < 1%,^4^^,^^5^ the response to treatment by these drugs in many patients is often slow resulting in treatment failure. In addition, relapse is reported in ∼10% of patients.^1^ *Burkholderia pseudomallei* can be persistent in the human host in the presence of antimicrobials and immune responses due to several adaptive mechanisms, for example, biofilm formation, intracellular invasion, phenotypic variation and acquired resistance to drugs.^6^^–^^10^

Because the current therapeutic options are limited, a new effective antimicrobial treatment is required for melioidosis. A new bacterial target is therefore needed to circumvent the preexisting antibiotic resistance mechanisms. One of the most interesting novel targets for the treatment of Gram-negative infections is lipid A biosynthesis. Lipid A biosynthesis is essential for the formation of lipopolysaccharide (LPS), a critical component of the Gram-negative outer membrane. Recent studies have demonstrated that many inhibitors of LpxC, the enzyme UDP-3-*O*-(R-3-hydroxymyristoyl)-*N*-acetylglucosamine deacetylase, responsible for the first step in lipid A biosynthesis, have potent bactericidal activity.^11^^–^^14^ The first study of an LpxC inhibitor activity against *B. pseudomallei* evaluated ACHN-975 with a small number of *B. pseudomallei* and other biodefense pathogens in vitro, and demonstrated MIC_50_ = 1 μg/mL and MIC_90_ = 2 μg/mL for *B. pseudomallei*.^15^ Among the LpxC inhibitors, LpxC-4 has been demonstrated to have superior activities against many Gram-negative pathogens compared with other LpxC inhibitors and meropenem. A recent study showed that LpxC-4 demonstrates bactericidal activity against *Pseudomonas aeruginosa*, *Klebsiella pneumoniae*, *Escherichia coli*, *Enterobacter* spp., *Burkholderia cepacia*, and *Stenotrophomonas maltophilia*.^14^ However, it is unknown whether the LpxC-4 is effective against all *B. pseudomallei* isolates.

The aim of this study was to test the in vitro activity of a novel inhibitor LpxC-4 against a large collection of *B. pseudomallei* isolates from Thai patients. The isolates showed different resistance profiles and included a CAZ/SXT–susceptible group, a CAZ-resistant group, and a SXT-resistant group. The synergistic activity of LpxC-4 combined with CAZ, and the effect of LpxC-4 on bacterial cell morphology, were also investigated. Evaluation of the antibacterial activity of LpxC-4 is needed to determine whether the LpxC inhibitor may be a promising new antibiotic to treat melioidosis.

## MATERIALS AND METHODS

### Bacterial isolates.

A total of 108 clinical *B. pseudomallei* isolates from our retrospective collections were tested. All experiments with *B. pseudomallei* were performed in a Biosafety Level 3 laboratory. These isolates were obtained from various clinical specimens of 108 melioidosis patients presented at Sappasitthiprasong Hospital, Ubon Ratchathani, Thailand, during 1986–2012. These included CAZ/SXT–susceptible (*N* = 71), CAZ-resistant (*N* = 14), and SXT-resistant (*N* = 23) *B. pseudomallei* isolates described in our previous studies.^4^^,^^5^^,^^16^ Reference strains used for susceptibility testing were *B. pseudomallei* K96243, *E. coli* ATCC 25922, and *P. aeruginosa* ATCC 27853. Isolates were stored in trypticase soy broth containing 15% glycerol at −80°C.

### Susceptibility testing.

Antimicrobial susceptibility to CAZ and SXT and minimum inhibitory concentration (MIC) data were obtained from our previous studies.^4^^,^^5^ Susceptibility testing for CAZ was performed using a disk diffusion test or E-test.^5^ Susceptibility to SXT was examined using an E-test.^4^ The MIC for CAZ was read at the 100% inhibition zone and for SXT was read at the 80% inhibition point.^4^^,^^5^^,^^17^ The MIC breakpoints used were as follows: CAZ, susceptible ≤ 8 μg/mL, intermediate 16 μg/mL, and resistant ≥ 32 μg/mL; SXT, susceptible ≤ 2/38 μg/mL and resistant ≥ 4/76 μg/mL. *Escherichia coli* ATCC 25922 and *P. aeruginosa* ATCC 27853 were used as controls for CAZ and SXT.^4^^,^^5^^,^^17^

Susceptibility to an LpxC inhibitor, LpxC-4, was examined using a broth microdilution test according to the Clinical and Laboratory Standards Institute guidelines.^17^ *Burkholderia pseudomallei* isolates were recovered from freezer vials by streaking onto Columbia agar and incubating aerobically for 24 hours at 37°C. Bacterial colonies were then harvested, suspended in normal saline, and adjusted to an optical density of 0.2 at 600 nm to obtain a concentration of 1 × 10^8^ colony-forming units (CFU)/mL. Bacteria at a final concentration of 5 × 10^5^ CFU/mL were used for susceptibility testing of LpxC-4 (catalog number PZ0194; Sigma-Aldrich, St. Louis, MO)^14^ at concentrations of 0, 0.5, 1, 2, 4, 8, 16, and 32 μg/mL in duplicate. The MIC was read as the lowest drug concentration at which no visible growth was observed after aerobic incubation at 37°C for 20 hours. To determine the minimum bactericidal concentration (MBC), 100 μL of the bacterial suspension was spread onto Columbia agar in triplicate to observe viability after aerobic incubation at 37°C for 20 hours. The MBC was read by determining the lowest concentration of LpxC-4 that reduced the viability of the initial bacterial inoculum by ≥ 99.9%.

### Time-kill assay.

*Burkholderia pseudomallei* K96243 was prepared as described for the susceptibility testing. Bactericidal activity of LpxC-4 against *B. pseudomallei* was assessed using a final concentration of bacteria of approximately 1 × 10^6^ CFU/mL in 5 mL of Mueller–Hinton broth (MHB) containing 2-fold serial dilutions of LpxC-4 (from 8 × to 0.5 × MIC, 16 μg/mL to 1 μg/mL). In a pilot study, the MIC for both LpxC-4 and CAZ against *B. pseudomallei* K96243 was 2 μg/mL. To investigate whether LpxC-4 has synergy with CAZ against *B. pseudomallei*, bactericidal activity was also assessed for CAZ alone at concentration of 4 × MIC (8 μg/mL), and LpxC-4 in combination with CAZ at 4 × MIC for each drug (8 μg/mL for LpxC-4 and 8 μg/mL for CAZ). The concentration at 4 × MIC was chosen because it represents levels of CAZ that are achievable in blood for long periods.^18^ A *B. pseudomallei* culture in MHB (Oxoid, Hants, United Kingdom) without antimicrobials was used as a control. One-hundred microliters of culture were collected 0, 2, 4, 6, 8, 10, and 24 hours postinoculation and incubation at 37°C with shaking at 200 rpm. The samples were serially diluted in normal saline, and 100 μL of bacterial suspension of each dilution was spread onto Columbia agar plates in triplicate. The plates were incubated aerobically overnight at 37°C for colony counting. Two independent experiments were performed and mean values were calculated. A bactericidal effect was defined as a ≥ 3 log_10_ CFU/mL decrease after 24 hours of incubation compared with the bacterial number of the initial inoculum. Synergism was defined as a decrease in the colony count of ≥ 2 log_10_ CFU/mL after exposure to the combination of drugs compared with the count obtained for the most active single drug.^19^

### Immunofluorescence staining.

*Burkholderia pseudomallei* K96243 was treated with LpxC-4 in MHB at a concentration of 8 μg/mL (4 × MIC) and was examined 0, 4, and 8 hours postinoculation and aerobic incubation at 37°C. For staining, 10 μL of *B. pseudomallei* was incubated with an equal volume of 4B11 monoclonal antibody–based immunofluorescent reagent (Mab-IFA),^20^^,^^21^ specific to *B. pseudomallei* exopolysaccharide,^22^ on a glass slide. A glass coverslip was placed over the top of the mixture, and the slide was incubated at room temperature for 10 minutes before observing the presence of green fluorescent bacteria using a fluorescence microscope at a 1,000× magnification (Olympus BH-2; Tokyo, Japan).

### Statistical analysis.

Statistical analyses were performed using Stata version 14.0 (StataCorp LP, College Station, TX). The Mann–Whitney test was used to test the difference between the medians of different *B. pseudomallei* groups. Spearman's rank correlation was performed to determine the correlation coefficient of the MICs between two *B. pseudomallei* groups. Differences were considered statistically significant if the *P* value was < 0.05.

## RESULTS

### Susceptibility of *B. pseudomallei* to LpxC inhibitor.

**Figure 1.**
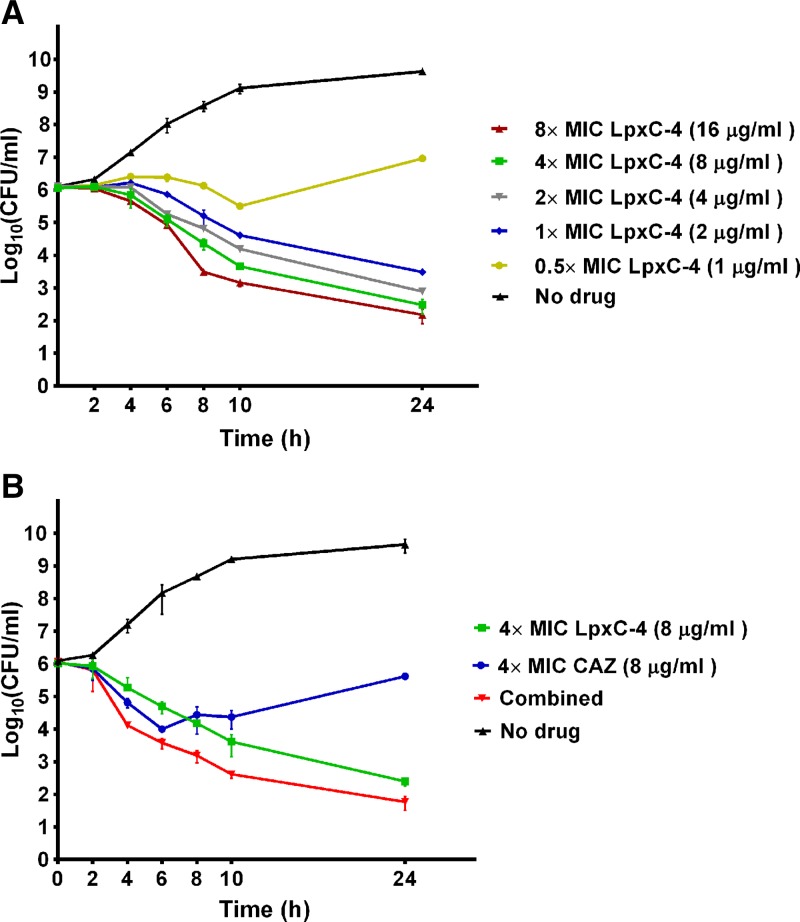
Time-kill curves for *Burkholderia pseudomallei* K96243. (**A**) LpxC-4 was tested at 0.5×, 1×, 2×, 4×, and 8× minimum inhibitory concentrations (MICs). (**B**) LpxC-4 and ceftazidime (CAZ) were tested individually at 4× MIC or in combination (4× MIC for each drug). Error bars represent standard deviation.

Time-kill kinetic experiments were performed using different concentrations of LpxC-4 against a reference strain, *B. pseudomallei* K96243. The results in Figure 1A

demonstrate the growth inhibition of *B. pseudomallei* at an LpxC-4 concentration of 1 × MIC (2 μg/mL) at 8, 10, and 24 hours, and bactericidal activity was detected at a drug concentration ≥ 2 × MIC (≥ 4 μg/mL) at 24 hours. LpxC-4 at 0.5 × MIC (1 μg/mL) showed growth inhibition at 10 hours, but regrowth was observed at 24 hours.

### Synergistic activity of LpxC inhibitor combined with CAZ.

Treatment with CAZ alone at 4 × MIC (8 μg/mL) showed an inhibitory effect against *B. pseudomallei* K96243 at 4 and 6 hours; however, significant bacterial regrowth was observed after 6 hours of incubation (Figure 1B). In contrast, regrowth was not observed after treatment with LpxC-4 alone at 4 × MIC. When the combination of LpxC-4 and CAZ was used, a bactericidal effect against *B. pseudomallei* was demonstrated at 10 hours and 24 hours. However, no significant synergistic effect was observed with the combination of LpxC-4 and CAZ when compared with the activity of LpxC-4 alone.

### Bactericidal effect of LpxC inhibitor on clinical isolates of *B. pseudomallei*.

**Figure 2.**
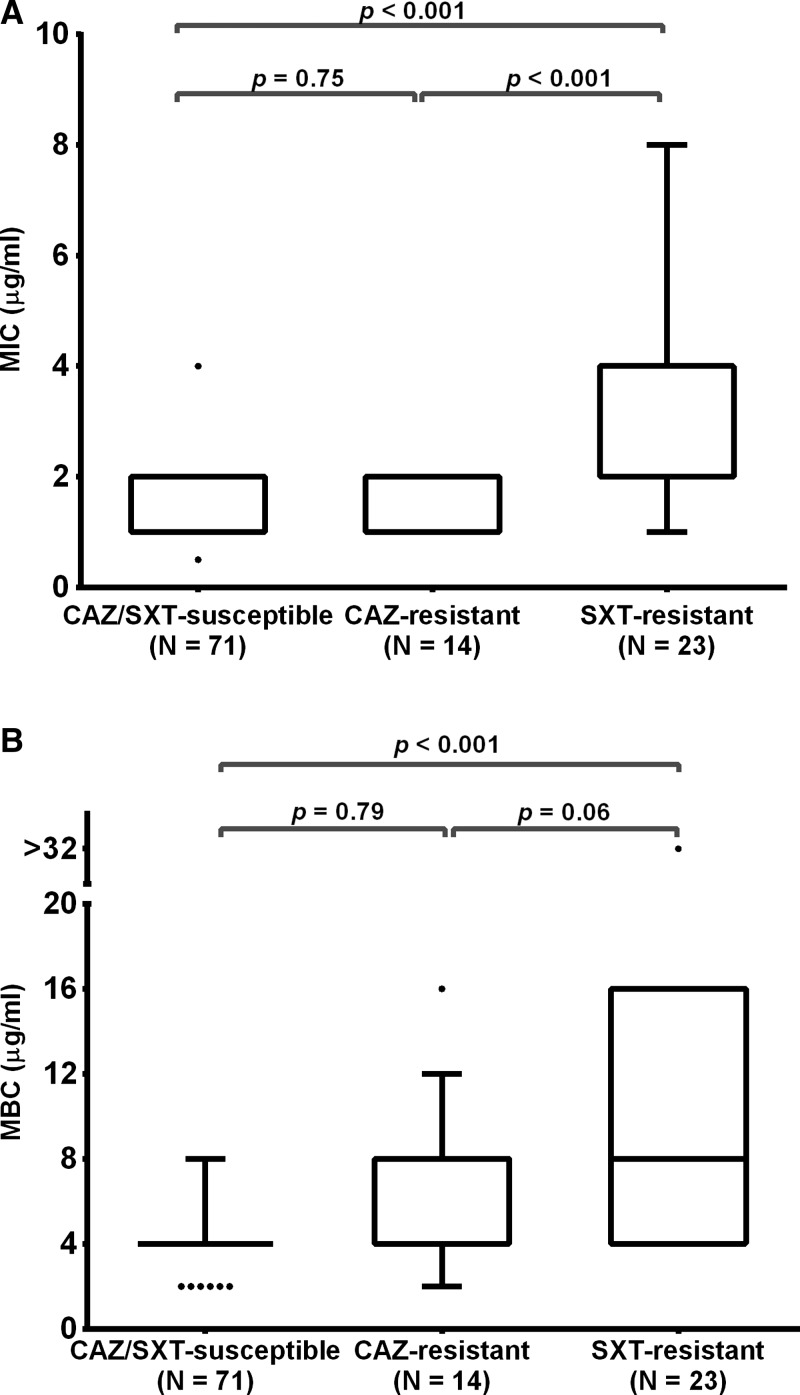
Susceptibility of LpxC-4 to three groups of *Burkholderia pseudomallei* isolates: ceftazidime (CAZ)/trimethoprim-sulfamethoxazole (SXT) susceptible, CAZ-resistant, and SXT resistant. Box plots represent the 25th and 75th percentile boundaries in the box, with the median line indicated within the box; the whiskers indicate the 10th and 90th percentiles. The plots show the (**A**) minimum inhibitory concentration (MIC) and (**B**) minimum bactericidal concentration (MBC) for each group of isolates.

Lipid A is a conserved molecule in *B. pseudomallei* (unpublished data). We examined whether LpxC-4 can kill clinical *B. pseudomallei* isolates. Because CAZ and SXT are drugs currently recommended for treatment of melioidosis patients, the bactericidal activity of LpxC-4 was determined in retrospective collections from 1986 to 2012, representing three groups of isolates: 1) CAZ/SXT susceptible (*N* = 71), 2) CAZ resistant (*N* = 14), and 3) SXT resistant (*N* = 23). The results are shown in Figure 2

and Supplemental Table 1. The LpxC-4 MIC required to inhibit the growth of 90% of microorganisms (MIC_90_) for all 108 isolates was 2 μg/mL. All isolates belonging to the CAZ/SXT–susceptible group were also susceptible to LpxC-4 (MIC ≤ 4 μg/mL and MBC ≤ 8 μg/mL). The median LpxC-4 MIC for this group was 1 μg/mL (interquartile range [IQR] = 1–2 μg/mL), which showed no significant difference when compared with the median LpxC-4 MIC of the CAZ-resistant group (median = 1 μg/mL, IQR = 1–2 μg/mL) (*P* = 0.75). However, the LpxC-4 MIC of the SXT-resistant group (median = 2 μg/mL, IQR = 2–4 μg/mL) was significantly higher than the MIC of the CAZ/SXT-susceptible group (*P* < 0.001).

The median MBC of LpxC-4 for the CAZ/SXT–susceptible group (median = 4 μg/mL, IQR = 4–4 μg/mL) showed no significant difference when compared with that of the CAZ-resistant group (median = 4 μg/mL, IQR = 4–8 μg/mL) (*P* = 0.79). However, the LpxC-4 MBC values of the SXT-resistant isolates varied between isolates (median = 8 μg/mL, IQR = 4–16 μg/mL) and were significantly higher than the LpxC-4 MBC of the CAZ/SXT–susceptible group (*P* < 0.001). These results suggest there might be an association between the resistance to LpxC-4 and the SXT resistance phenotype. We determined whether the LpxC-4 MIC value correlated with the SXT MIC value. However, pairwise correlations of the MIC values for all 23 SXT-resistant isolates demonstrated a low relatedness between the MIC of LpxC-4 and SXT resistance (correlation coefficient, rho = 0.33).

### Effect of LpxC-4 on *B. pseudomallei* morphology.

**Figure 3.**
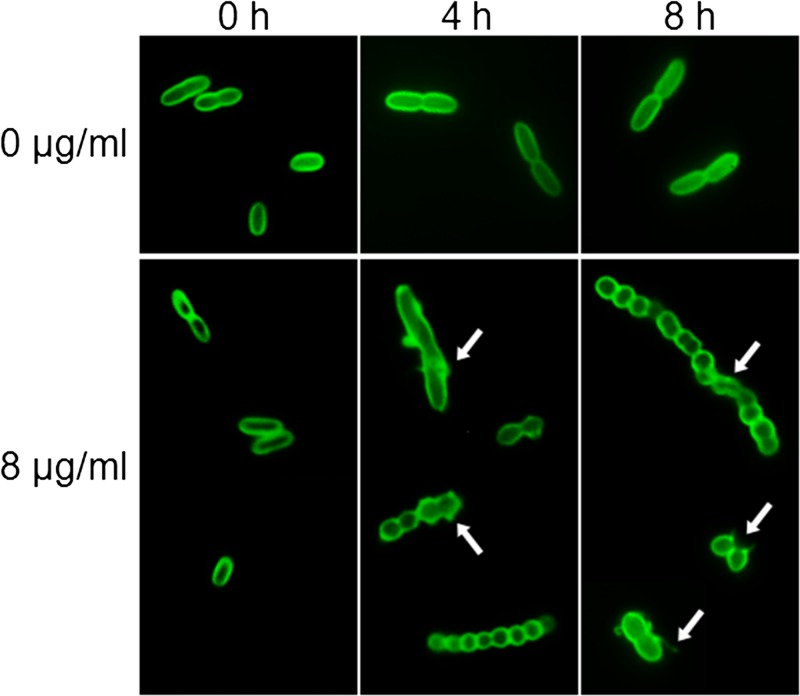
Immunofluorescence staining of *Burkholderia pseudomallei* K96243 cells after treatment with 8 μg/mL of LpxC-4 for 0, 4, and 8 hours; 0 μg/mL of LpxC-4 was included as a control. Arrows indicate bacterial cells with cell surface damage.

LpxC-4 potentially exerts bactericidal activity against *B. pseudomallei* by inhibition of lipid A biosynthesis. We observed the morphology of *B. pseudomallei* cells after exposure to 8 μg/mL of LpxC-4 by immunofluorescent staining. The experiments were performed using strain K96243 and three LpxC-4 resistant isolates with an LpxC-4 MIC ≥ 8 μg/mL (H2732a, H4697a, and H5598a) (Supplemental Table 1). All *B. pseudomallei* K96243 cells showed morphological changes from a bacillus form to a spherical form at 4 and 8 hours after drug treatment (Figure 3

). Surviving bacteria were arranged in chains, suggested the failure of cell division. Many *B. pseudomallei* cells showed areas of surface damage. Few bacteria were detected at 10-hour incubation time, and none were detected at 24 hours, which was the time point associated with cell death (Figure 1). The morphology of the three LpxC-4-resistant isolates showed a mixed population of typical rod and spherical forms at 4 and 8 hours (data not shown).

## DISCUSSION

Despite the reported low rate of antimicrobial resistance to CAZ in vitro,^4^^,^^5^ the treatment response to this drug in melioidosis cases is not completely understood. In northeast Thailand, death occurred in 40% of patients who received treatment. Smith and others showed that CAZ was not bactericidal for *B. pseudomallei* strain 576a and five other strains, and significant bacterial regrowth could occur at 24 hours.^18^ The development of CAZ resistance among clinical isolates during treatment has been described previously.^5^^,^^6^^,^^23^ Resistance could be mediated by deletion of the penicillin-binding protein 3 target via large genomic deletions,^6^ or by mutations affecting the expression and structure of chromosomally encoded PenA β-lactamase.^23^ Our results confirmed the potential activity of the inhibitor of lipid A biosynthesis enzyme for the treatment of melioidoisis.^15^ Our study demonstrated the LpxC-4 was effective against a large number of *B. pseudomallei* isolates, including CAZ-resistant isolates. Our data suggest that the mechanisms that mediate resistance to CAZ do not contribute to LpxC-4 resistance in *B. pseudomallei*. The finding that 100% of isolates belonging to the CAZ/SXT–susceptible and CAZ-resistant groups were susceptible to LpxC-4 was comparable with previously reported rates of susceptibility to CAZ (99.8%).^4^^,^^5^ LpxC-4 was also studied in combination with CAZ, but it had no synergistic effect on the action of CAZ.

Lipid A is the anchor for LPS on the outer membrane of Gram-negative bacteria; therefore, the synthesis of lipid A molecules is of vital importance among the various components that are responsible for outer membrane assembly. A small-molecule inhibitor that interferes with lipid A biosynthesis, such as LpxC-4, can inhibit LPS assembly resulting in damage and the rapid death of target cells.^14^ Indeed, we observed the loss of integrity of the cell structure of *B. pseudomallei* after treatment with LpxC-4. Fluorescent microscopy of LpxC-4-treated *B. pseudomallei* cells stained with Mab-IFA reagent revealed chains of undividing spherical bacterial cells. The conversion from rod-shaped cells to viable cell surface–defective spherical cells has also been observed when *P. aeruginosa* was treated with penicillin and carbapenems.^24^

Spontaneous resistance to LpxC-4 was found in several *B. pseudomallei* isolates in the SXT-resistant group. Our data demonstrated that the SXT-resistant group had a significantly increased LpxC-4 MIC. The mechanism of SXT resistance in *B. pseudomallei* has been reported to involve efflux pump expression, which may also be implicated in resistance to other drugs,^8^^,^^23^ including LpxC-4. Three efflux pumps have been characterized in *B. pseudomallei*, namely AmrAB-OprA, BpeAB-OprB, and BpeEF-OprC, but BpeEF-OprC is clinically most significant and widespread in many Australian and Thai isolates.^8^^,^^23^ In addition, other mechanisms for drug resistance in *B. pseudomallei*, such as biofilm production^8^^,^^25^^,^^26^ and outer membrane impermeability,^8^^,^^23^ may involve in the resistance to LpxC-4. Biofilm formation has previously been linked to resistance to CAZ and meropenem (MEM).^25^^,^^26^

Tomaras and others reported that different Gram-negative pathogens can use different mechanisms of resistance to LpxC inhibitors. These include overexpression of efflux systems, regulation of *lpxC* expression levels, and mutation of the *fabZ* gene that could affect lipid A and fatty acid biosynthesis.^14^ It is possible that *B. pseudomallei* isolates can use any of these LpxC-4 resistance mechanisms. In animal models of infection, LpxC-4 has been shown to be efficacious against *P. aeruginosa* and *K. pneumoniae*.^14^ Activity in these models was correlated with MIC values of the strains evaluated and pharmacokinetic/pharmacodynamic driver analysis suggests the area under the concentration–time curve/MIC to be the parameter linked to efficacy. A clinical dose of approximately 1,200 mg every 8 hours has been predicted to treat a few strains of *P. aeruginosa* and *K. pneumoniae* with MICs of 1 μg/mL. It is unknown whether the LpxC-4 can achieve a final concentration in the human blood of 1 μg/mL and shows no toxicity. The factors involved in the resistance to LpxC inhibitor in *B. pseudomallei* and the toxicity of the drug remain to be investigated.

In conclusion, we demonstrated that LpxC-4 is an effective antimicrobial against clinical isolates of *B. pseudomallei.* LpxC enzyme should therefore be considered for further evaluation of its in vivo efficacy and toxicity. The future application of an inhibitor of lipid A biosynthesis as a novel antibiotic target for the treatment of melioidosis is promising.

## Supplementary Material

Supplemental Table.
