# Supplementary material for: Susceptibility of Clinical Isolates of Burkholderia pseudomallei to a Lipid A Biosynthesis Inhibitor
Source: Am J Trop Med Hyg. 2017 Apr 24;97(1):62–7. doi: 10.4269/ajtmh.16-0858 (PMC5508901; doi:10.4269/ajtmh.16-0858)
Supplement: Supplementary file 1 [file tpmd160858.SD1.pdf]

Supplemental Table 1

Susceptibility of *Burkholderia pseudomallei* to LpxC inhibitor, CAZ, and SXT

Group	Strain	Date of isolation	Specimen type	CAZ MIC (µg/mL)*	SXT MIC (µg/mL)†	LpxC-4 MIC (µg/mL)	LpxC-4 MBC (µg/mL)
CAZ/SXT susceptible (*N* = 71)	H206a	22/10/1986	Blood	ND	≤ 2	1	4
	H226a	19/12/1986	Wound swab	ND	≤ 2	1	8
	H257a	02/07/1987	Pus	ND	≤ 2	1	2
	H303b	26/08/1987	Blood	ND	≤ 2	1	4
	H423a	04/10/1988	Blood	ND	≤ 2	1	8
	H428a	18/10/1988	Sputum	ND	≤ 2	1	4
	H430e	22/10/1988	Blood	ND	≤ 2	1	4
	H436b	02/11/1988	Blood	ND	≤ 2	2	4
	H443a	10/12/1988	Pleural fluid	ND	≤ 2	1	4
	H448a	20/12/1988	Blood	ND	≤ 2	1	4
	H479a	08/06/1989	Sputum	ND	≤ 2	1	4
	H480a	07/06/1989	Lung	ND	≤ 2	1	8
	H504a	16/07/1989	Blood	ND	≤ 2	1	4
	H596a	27/10/1989	Blood	ND	≤ 2	1	4
	H646a	03/05/1990	Sputum	ND	≤ 2	2	8
	H757a	11/12/1990	Liver aspirate	ND	≤ 2	1	4
	H763a	05/01/1991	Pleural fluid	ND	≤ 2	1	8
	H770b	25/01/1991	Sputum	ND	≤ 2	2	8
	H778a	23/03/1991	Liver aspirate	ND	≤ 2	1	4
	H845a	08/09/1991	Rectal swab	ND	≤ 2	2	4
	H855e	05/10/1991	Rectal swab	ND	≤ 2	1	4
	H1066a	15/07/1993	Liver aspirate	ND	≤ 2	1	4
	H1119a	14/09/1993	Pleural fluid	ND	≤ 2	2	8
	H1143a	08/10/1993	Liver aspirate	ND	≤ 2	1	4
	H1165a	30/10/1993	Sputum	ND	≤ 2	1	4
	H1189a	15/01/1994	Liver aspirate	ND	≤ 2	1	4
	H1210a	07/05/1994	Blood	ND	≤ 2	1	4
	H1219a	26/05/1994	Wound swab	ND	≤ 2	0.5	2
	H1234a	30/06/1994	Urine	ND	≤ 2	1	4
	H1237a	01/07/1994	Blood	ND	≤ 2	2	8
	H1303b	08/10/1994	Blood	ND	≤ 2	1	4
	H1304a	11/10/1994	Liver aspirate	ND	≤ 2	1	4
	H1338a	18/12/1994	Liver aspirate	ND	≤ 2	2	8
	H1400b	16/07/1995	Blood	ND	≤ 2	1	4
	H1401a	12/07/1995	Blood	ND	≤ 2	1	4
	H1463a	04/09/1995	Blood	ND	≤ 2	1	4
	H1479a	21/09/1995	Urine	ND	≤ 2	1	4
	H1494a	13/10/1995	Wound swab	ND	≤ 2	1	4
	H1495a	13/10/1995	Splenic aspirate	ND	≤ 2	1	4
	H1501a	28/10/1995	Wound swab	ND	≤ 2	1	4
	H1577a	03/07/1996	Sputum	ND	≤ 2	1	4
	H1620a	05/09/1996	Blood	ND	≤ 2	1	4
	H1626a	11/09/1996	Urine	ND	≤ 2	1	4
	H1628b	20/09/1996	Blood	ND	≤ 2	2	8
	H1634a	20/09/1996	Blood	ND	≤ 2	2	8
	H1660a	18/10/1996	Pleural fluid	ND	≤ 2	2	4
	H1686a	13/11/1996	Blood	ND	≤ 2	2	4
	H1690a	22/11/1996	Blood	ND	≤ 2	2	4
	H1698a	12/12/1996	Liver aspirate	ND	≤ 2	2	4
	H1699a	13/12/1996	Pus	ND	≤ 2	2	4
	H1790a	25/07/1997	Throat swab	ND	≤ 2	2	4
	H1897a	18/12/1997	Blood	ND	≤ 2	1	4
	H1923a	18/03/1998	Sputum	ND	≤ 2	2	4
	H2029b	07/08/1998	Blood	ND	≤ 2	2	4
	H2033a	07/08/1988	Wound swab	ND	≤ 2	2	4
	H2226a	07/03/1999	Blood	ND	≤ 2	1	4
	H2310a	16/07/1999	Blood	ND	≤ 2	4	8
	H2507a	23/06/2000	Pus	ND	≤ 2	2	8
	H2622a	27/02/2001	Blood	ND	≤ 2	2	4
	H2624a	06/03/2001	Blood	ND	≤ 2	2	8
	H2644a	27/05/2001	Blood	ND	≤ 2	2	4
	H2817a	25/03/2002	Blood	ND	≤ 2	2	2
	H2821a	30/05/2002	Blood	ND	≤ 2	2	4
	H2831a	19/06/2002	Sputum	ND	≤ 2	2	2
	H2944b	15/10/2002	Blood	ND	≤ 2	2	8
	H2991b	31/05/2003	Blood	ND	≤ 2	2	4
	H3161a	07/10/2003	Wound swab	ND	≤ 2	1	4
	H3171a	14/10/2003	Blood	ND	≤ 2	2	4
	H3183a	25/10/2003	Splenic aspirate	ND	≤ 2	1	2

(*continued*)

Supplemental Table 1
Continued

Group	Strain	Date of isolation	Specimen type	CAZ MIC (μg/mL)*	SXT MIC (μg/mL)†	LpxC-4 MIC (μg/mL)	LpxC-4 MBC (μg/mL)
	H3192a	08/11/2003	Blood	ND	≤ 2	1	2
	H3299a	26/06/2004	Lung	ND	≤ 2	1	4
CAZ resistant (*N* = 14)	H316c	14/11/1987	Blood	48	3	2	8
	H402g	31/08/1988	Blood	48	2	1	2
	H490f	05/07/1989	Wound swab	512	0.75	1	4
	H533eii	08/09/1989	Sputum	512	2	1	4
	H577ci	16/10/1989	Sputum	512	0.19	1	4
	H858fi	29/10/1991	Blood	64	0.5	1	4
	H942dii	27/07/1992	Wound swab	64	1.5	1	4
	H956c	20/08/1992	Blood	48	2	1	2
	H975d	27/09/1992	Sputum	32	1	2	4
	H979bii	17/10/1992	Tracheal suction	512	6	1	4
	H2690a	07/08/2001	Sputum	512	0.75	2	16
	H3013c	14/06/2003	Sputum	32	1.5	2	8
	H3964d	20/08/2006	Blood	64	1.52	2	8
	H4095c	11/10/2006	Pleural fluid	48	2	1	4
SXT resistant (*N* = 23)	H962a	22/08/1992	Sputum	ND	6	2	4
	H1051a	22/06/1993	Wound swab	ND	6	1	4
	H1373a	31/05/1995	Liver aspirate	ND	≥ 32	4	8
	H1374a	31/05/1995	Lymph node	ND	≥ 32	2	8
	H1375a	05/06/1995	Pleural fluid	ND	≥ 32	4	16
	H1468a	11/09/1995	Sputum	ND	≥ 32	2	4
	H1482a	24/09/1995	Blood	ND	4	2	8
	H1664a	24/10/1995	Sputum	ND	≥ 32	1	4
	H1713a	10/02/1997	Urine	ND	≥ 32	2	8
	H2069a	23/08/1998	Throat swab	ND	≥ 32	2	8
	H2412a	04/11/1999	Blood	ND	6	2	4
	H2732a	04/10/2001	Sputum	ND	≥ 32	8	16
	H2915a	12/09/2002	Sputum	ND	6	2	4
	H3532a	05/04/2005	Sputum	ND	≥ 32	2	4
	H3925a	16/07/2006	Blood	ND	6	2	4
	H4260a	17/03/2007	Pus	ND	16	1	4
	H4263a	23/03/2007	Sputum	ND	≥ 32	2	4
	H4697a	04/12/2007	Sputum	ND	≥ 32	8	≥ 32
	H5103a	17/10/2008	Blood	ND	≥ 32	4	8
	H5598a	20/05/2010	Blood	ND	12	8	16
	HBPUB10370A	23/03/2011	Sputum	ND	6	2	4
	HBPUB10497A	18/07/2011	Sputum	ND	≥ 32	4	16
	HBPUB10336E	06/06/2012	Sputum	ND	≥ 32	2	16

CAZ = ceftazidime; MBC = minimum bactericidal concentration; MIC = minimum inhibitory concentration; ND = not determined; SXT = trimethoprim–sulfamethoxazole. Susceptibility to LpxC-4 was tested against three groups of *B. pseudomallei* isolates: 1) CAZ/SXT susceptible (*N* = 71), 2) CAZ resistant (*N* = 14), and 3) SXT resistant. Susceptibility to CAZ was determined using a disk diffusion test or E-test. Susceptibility to SXT was determined using an E-test. The MIC breakpoints used were as follows: CAZ, susceptible ≤ 8 μg/mL, intermediate 16 μg/mL, and resistant ≥ 32 μg/mL; SXT, susceptible ≤ 2/38 μg/mL and resistant ≥ 4/76 μg/mL.

* ND, CAZ MIC was not determined using an E-test, but susceptibility was assessed by a disc diffusion assay.

† Detailed SXT MIC of the CAZ/SXT-susceptible group was not available. An E-test was performed but the susceptibility was reported to be ≤ 2 μg/mL.
